# In silico analysis of a chimeric fusion protein as a new vaccine candidate against *Clostridium perfringens* type A and *Clostridium septicum* alpha toxins

**DOI:** 10.1007/s00580-020-03136-6

**Published:** 2020-07-14

**Authors:** Ali Haghroosta, Hossein Goudarzi, Ebrahim Faghihloo, Zohreh Ghalavand, Mohammad Mahdi Ranjbar, Reza Pilehchian Langroudi

**Affiliations:** 1 Department of Microbiology, School of Medical, Shahid Beheshti University of Medical Sciences, Koodak-yar St, Daneshjoo Blvd, Velenjak, Chamran HWY, Tehran, Iran; 2 Department of Anaerobic Bacterial Vaccines Production, Razi Vaccine and Serum Research Institute, Agricultural Research, Education and Extension Organization (AREEO), Karaj, Iran; 3 Department of Poultry Virology Research, Razi Vaccine and Serum Research Institute, Agricultural Research, Education and Extension Organization (AREEO), Karaj, Iran

**Keywords:** *Clostridium perfringens*, *Clostridium septicum*, Protein fusion, Vaccine, In silico

## Abstract

In silico analysis is the most important approach to understand protein structure and functions, and the most important problem for designing and producing a fusion construct is producing large amounts of functional protein. *Clostridium perfringens* type A and *Clostridium septicum* produce alpha (*plc*) and alpha toxins respectively. *C. perfringens* can cause gas gangrene and gastrointestinal diseases. *C. septicum* can cause traumatic and non-traumatic gas gangrene. The aim of current research was in silico analysis of a chimeric fusion protein against *C. perfringens* type A and *C. septicum* alpha toxins. Firstly, the chimeric fusion gene was designed according to nucleotide sequences of *C. perfringens* type A alpha (KY584046.1) and *C. septicum* alpha (JN793989.2) toxin genes and then its fusion protein is constructed by amino acid sequences of *C. perfringens* type A and *C. septicum* alpha toxins. Secondly, online software was used to determine prediction of secondary and tertiary structures and physicochemical characteristics of the fusion protein. Finally, the validation of the fusion protein was confirmed by Rampage and proSA program. The designed fusion protein has 777 amino acids in length. TASSER server and physicochemical parameters are showed: C-score = − 2.68 and molecular weight = 87.9 KD respectively. Rampage and proSA software revealed the fusion protein is valid. Deposited accession number for the sequence of the fusion gene in the GenBank is MK908396. The designed fusion protein is valid and functional. Thus, the fusion gene could be used for clone and expression in a proper prokaryotic cell and also as a recombinant vaccine candidate.

## Introduction

*Clostridium perfringens* is a Gram-positive, anaerobic, spore-forming bacteria. It causes anaerobic cellulitis, myonecrosis (gas gangrene), enteritis necroticans, and food poisoning in humans and gastrointestinal and enterotoxemic diseases in other animals (Dwivedi et al. 2015). *C. perfringens* based on the production of 4 major lethal toxins such as alpha, beta, epsilon, and iota is classified into 5 toxinotype (A, B, C, D, E) (Petit et al. 1999). One of the most important lethal and dermonecrotic toxins is alpha toxin (phospholipase C) produced in different amounts by all *C. perfringens* types (A–E), the predominant product in *C. perfringens* type A, stated as a primary virulence factor implicated in the necrotic enteritis and gas gangrene (Sakurai et al. 2004). The *C. perfringens* type A alpha toxin gene(*cpa*) is located on a chromosome and is 1197 bp in length, and translated into a mature protein containing 398 aa, with a molecular weight of 43 kDa (Takahashi et al. 1974; Saint-Joanis et al. 1989). Alpha toxin has phospholipase C(PLC) activity, is a zinc metalloenzyme, and can connect to target cell membranes in the presence of Ca^2+^ ions. The three-dimensional structure of the toxin by X-ray crystallography displays a two-domain protein. The N-terminal domain (residues 1–246) is a predicted structure similar to *Bacillus cereus* phospholipase C, and the C-terminal (residues 256–370) is a structure similar to eukaryotic Ca^2+^-binding C2 domains and also a linker fragment (residues 247–255) that binds the 2 domains (Naylor et al. 1998). During the twentieth century, several researchers showed that immunization could be used to prevent myonecrosis (Dwivedi et al. 2015). In 2012, Langroudi et al. investigated in silico analysis of a fusion protein against *C. perfringens* type D and B toxins and showed that the fusion protein is functional (Langroudi et al. 2012). *C. septicum* is a Gram-positive, anaerobic, spore-forming, motile bacteria. It is the causative agent of traumatic gas gangrene and non-traumatic gas gangrene in patients with various diseases that affect the colon, necrotizing enterocolitis, and pericarditis (Gordon et al. 1997). Several toxins such as alpha, beta, gamma, and delta are produced by *C. septicum*. One of the most important lethal virulence factors of *C. septicum* is alpha toxin (AT) and responsible for myonecrosis. This toxin utilizes its pathogenesis by pore formation on the host cell surface and also by a range of effects on the target cell (Tweten 2001). Secreted AT is an inactive protoxin and proteolytic hydrolysis is required for its activation. Protoxin located on the target cell membrane and connected to the GPI-anchored proteins (Ballard et al. 1993; Knapp et al. 2010). The *Clostridium septicum* alpha toxin gene (*csa*) is located on a chromosome and is 1332 bp in length and translated into an inactive protoxin protein containing 443 aa with a molecular weight of 48 kDa. The 48 kDa toxin via a carboxy-terminal cleavage site (located 4 kDa from the carboxy-terminus) was activated by trypsin (Imagawa et al. 1994; Ballard et al. 1993). The three-dimensional structure of the AT toxin by X-ray crystallography shows that AT structure, and sequence is similar to large lobe (D2-D4) of aerolysin. Because aerolysin is divided into 2 lobe proteins where the small lobe is domain D1 and the large lobe is domains D2–D4, respectively (Melton-Witt et al. 2006). In 2018, a protein fusion was developed against *C. perfringens* type D epsilon and *C. septicum* alpha toxins (Kamalirousta and Pilehchian 2018). The objective of current research was in silico analysis of a chimeric fusion protein against *C. perfringens* type A and *C. septicum* alpha toxins.

## Materials and methods

### Designing alpha-alpha fusion gene and protein

*Clostridium perfringens* alpha toxin gene complete cds KY584046.1 and *csa* gene complete cds JN793989.2 were retrieved from the NCBI database (Saint-Joanis et al. 1989; Imagawa et al. 1994). In the new construction, *cpa* and *csa* genes are linked together via the linker fragment A(EAAAK)_2_A (Chen et al. 2013). At the 5′ end of *cpa* and the 3′ end of *csa* respectively was added *NdeI* and *XhoI* restriction sites and their flanking regions, and then α-α fusion protein is constructed by nucleotide sequences of *cpa* and *csa* genes.

### Prediction of alpha-alpha fusion protein structure by online software

InterproScan program was used for prediction of patterns and profiles of the fusion protein (http://ebi.ac.uk/Tools/InterproScan) (Jones et al. 2014). Secondary structure was predicted using proMotif program of proFunc server (http://www.ebi.ac.uk/thornton-srv/databases/pdbsum) (Laskowski et al. 2005a, b). The prediction of linker fragment and the transmembrane helix locations in the fusion protein were carried out by GOR4 and HMMTOP (http://npsa-prabi.ibcp.fr/cgi-bin/secpred_gor4.p1 and http://www.enzym.hu/hmmtop/server/hmmtop.cgi), respectively (Combet et al. 2000; Tusnady and Simon 2001). Signal peptide cleavage sites were predicted using SignalP (Petersen et al. 2011). Tertiary structure of the fusion protein was predicted using I-TASSER server (www.zhanglab.ccmb.med.umich.edu/I-TASSER) (Yang et al. 2015; Roy et al. 2010; Zhang 2008) and then was visualized by UCSF Chimera software (www.rbvi.ucsf.edu/chimera) (Pettersen et al. 2004; Chen et al. 2014; Goddard et al. 2007). The ProtParam program was used to identify the physicochemical characteristics of the fusion protein (http://web.expasy.org/cgi-bin/protparam) (Gasteiger et al. 2005).

### Prediction of the alpha-alpha fusion protein binding sites

Prediction of the construct fusion protein binding sites carried out by Phyre^2^ online software (Kelley et al. 2015; Wass et al. 2010). The total translated fusion protein residues of 777 aa uploaded. Three templates such as c3c0mB.pdb, d3c0na2.pdb, and d1uyja.pdb were chosen to model the construct fusion protein based on heuristics to maximize confidence, percentage identity, and alignment coverage.

### Energy minimize of the fusion protein and its validation

The model was exposed for energy minimizing by SPDBV_4.10 software (http://www.expasy.org/spdbv) (Guex and Peitsch 1997), and then Rampage (http://mordred.bioc.cam.ac.uk/~rapper/rampage.php) and proSA

(http://prosa.services.came.sbg.ac.at/prosa.php) software was used for validating geometrical structure as a natural like protein (Lovell et al. 2003; Wiederstein and Sippl 2007; Sippl 1993).

## Results

### Construct of the fusion gene and protein

**Fig. 1:**
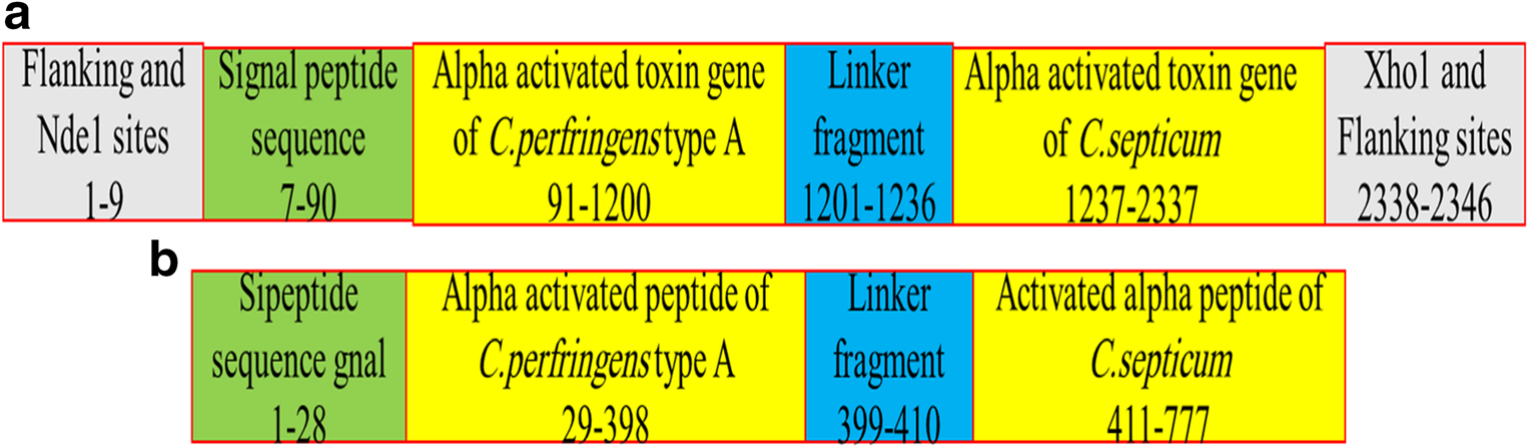
**A** Schematic view of the designed synthetic construct fusion gene. **B** Schematic view of the α-α chimeric fusion protein

The α-α fusion gene construct was 2346 bp in length and includes *cpa* (nucleotides1–1200), linker (nucleotides12001–1236) as GCGGAAGCGGCGGCGAAAGAAGCGGCGGCGAAAGCG, and nucleotides 1237 to 2346 that belonged to *csa* gene, (Fig. 1A and B).

### Prediction of the fusion protein structure by online software

**Fig. 2:**
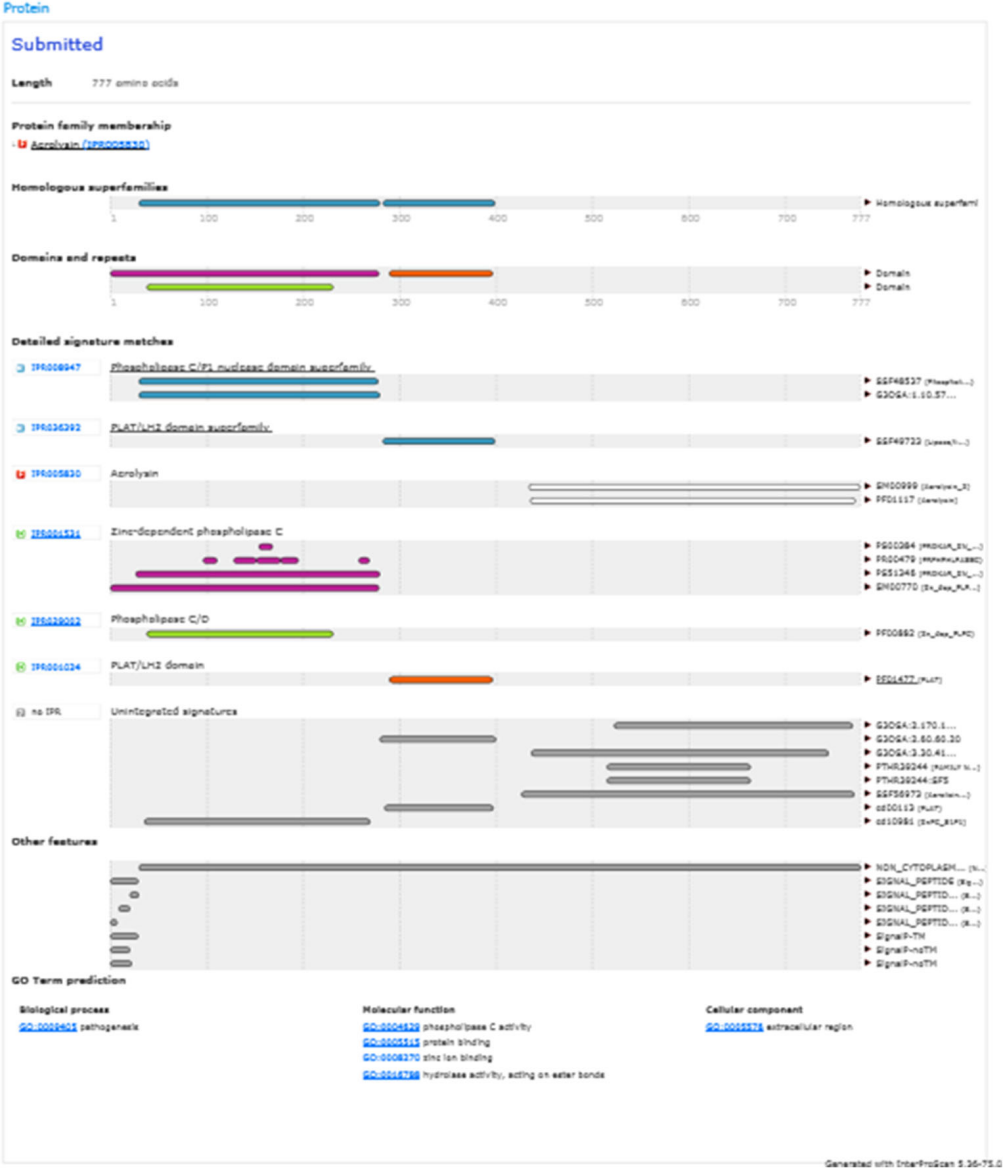
InterproScan for α-α fusion chimeric protein

**Fig. 3:**
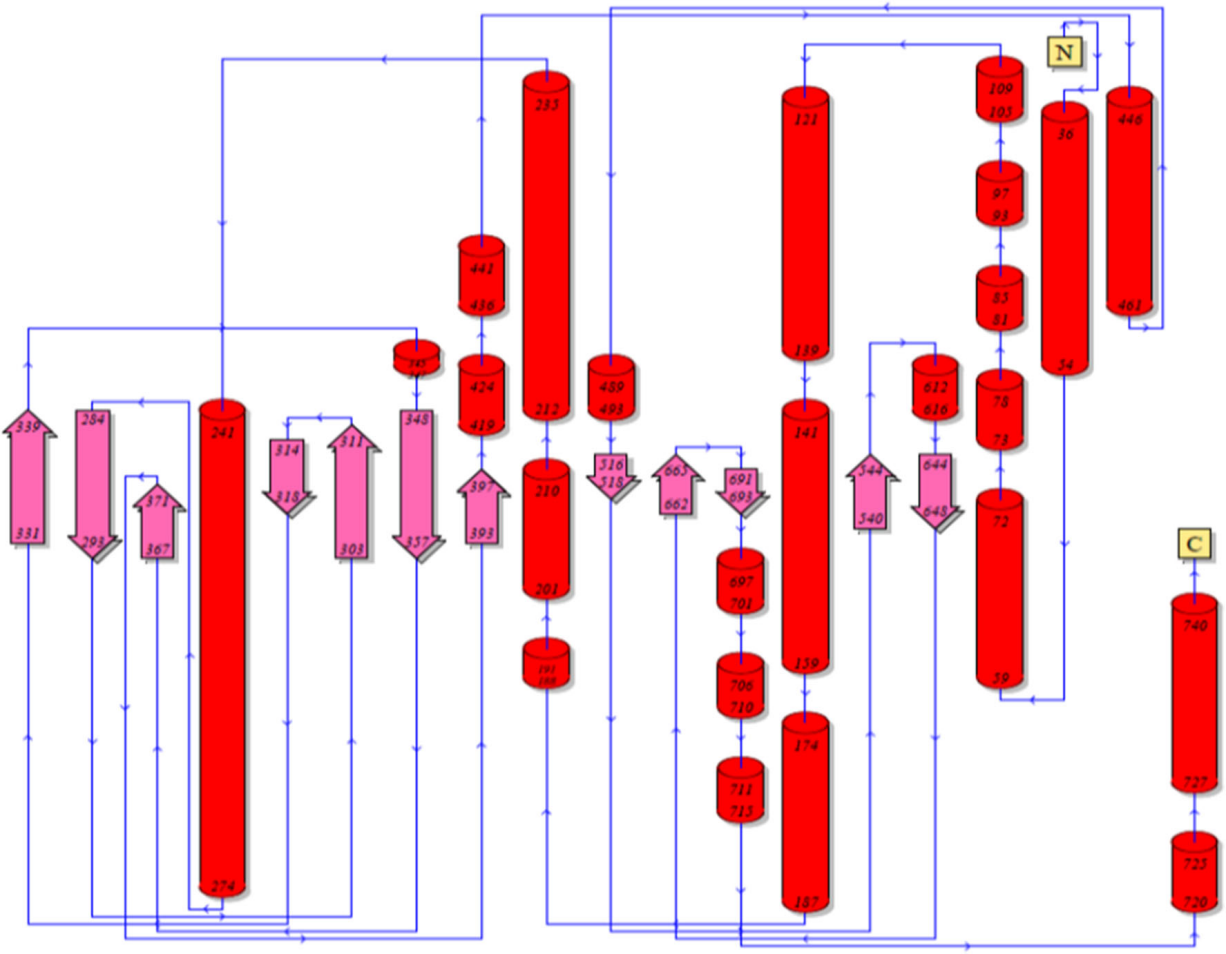
Secondary structure of fusion protein construction by ProMotif program of ProFunc server

**Fig. 4:**
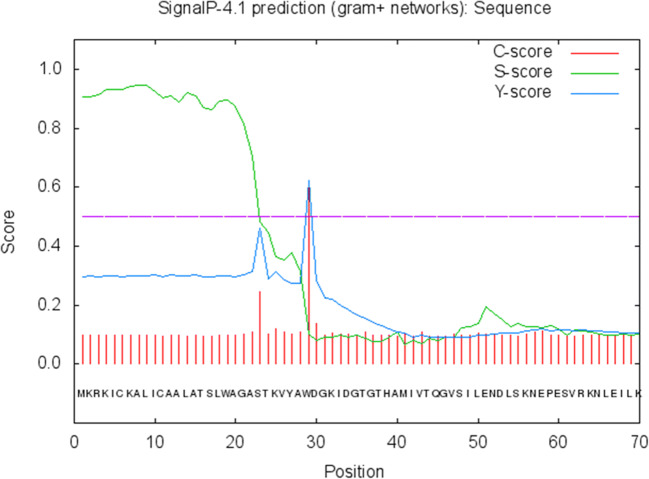
Prediction of signal peptide cleavage sites

**Fig. 5:**
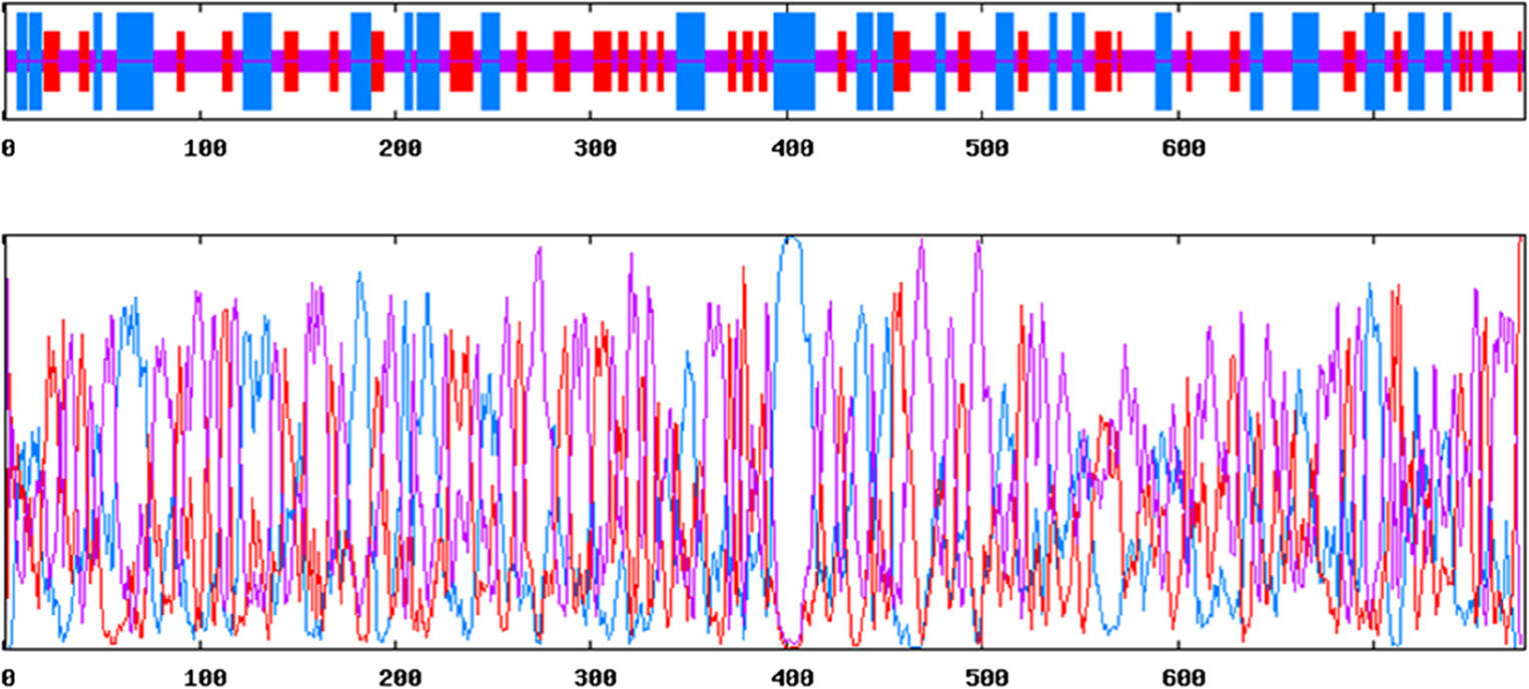
Linker fragment location in the fusion protein by GOR

**Fig. 6:**
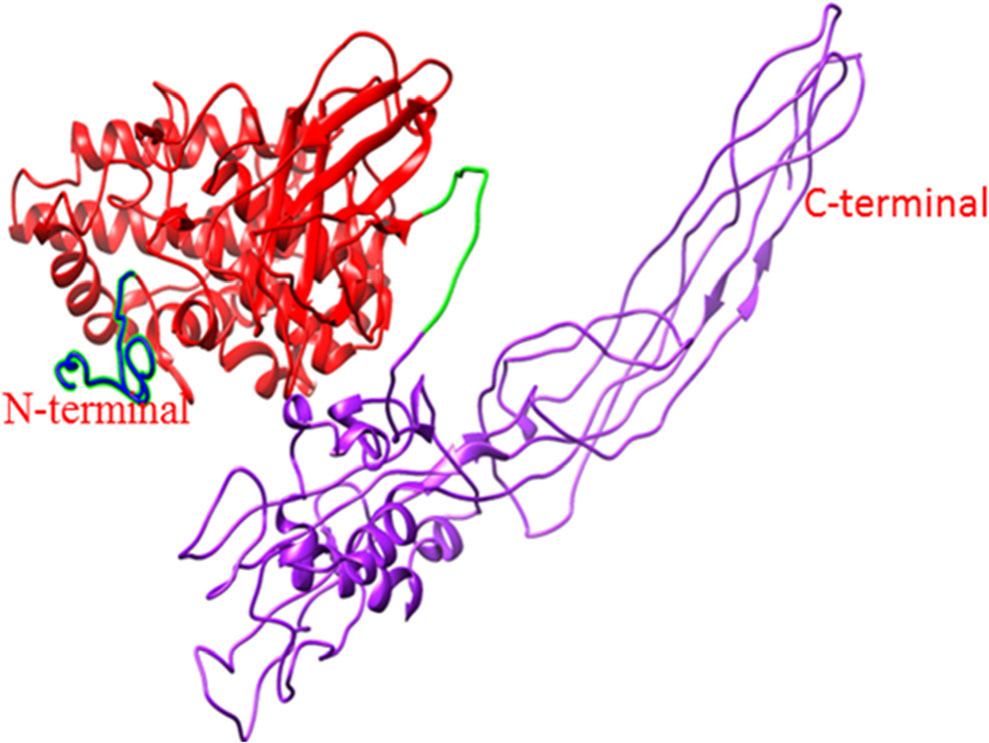
Informative 3D Visualization of predicted alpha-alpha fusion protein tertiary structure by I-TASSER. Blue color: Signal peptide of *C. perfringense* alpha toxin(amino acids1–28); red color: *C. perfringense* mature alpha toxin (amino acids 29–398); Green color: Linker (amino acids 399–410); purple color: *C. septicum* activated alpha toxin (amino acids 411–777)

**Fig. 7:**
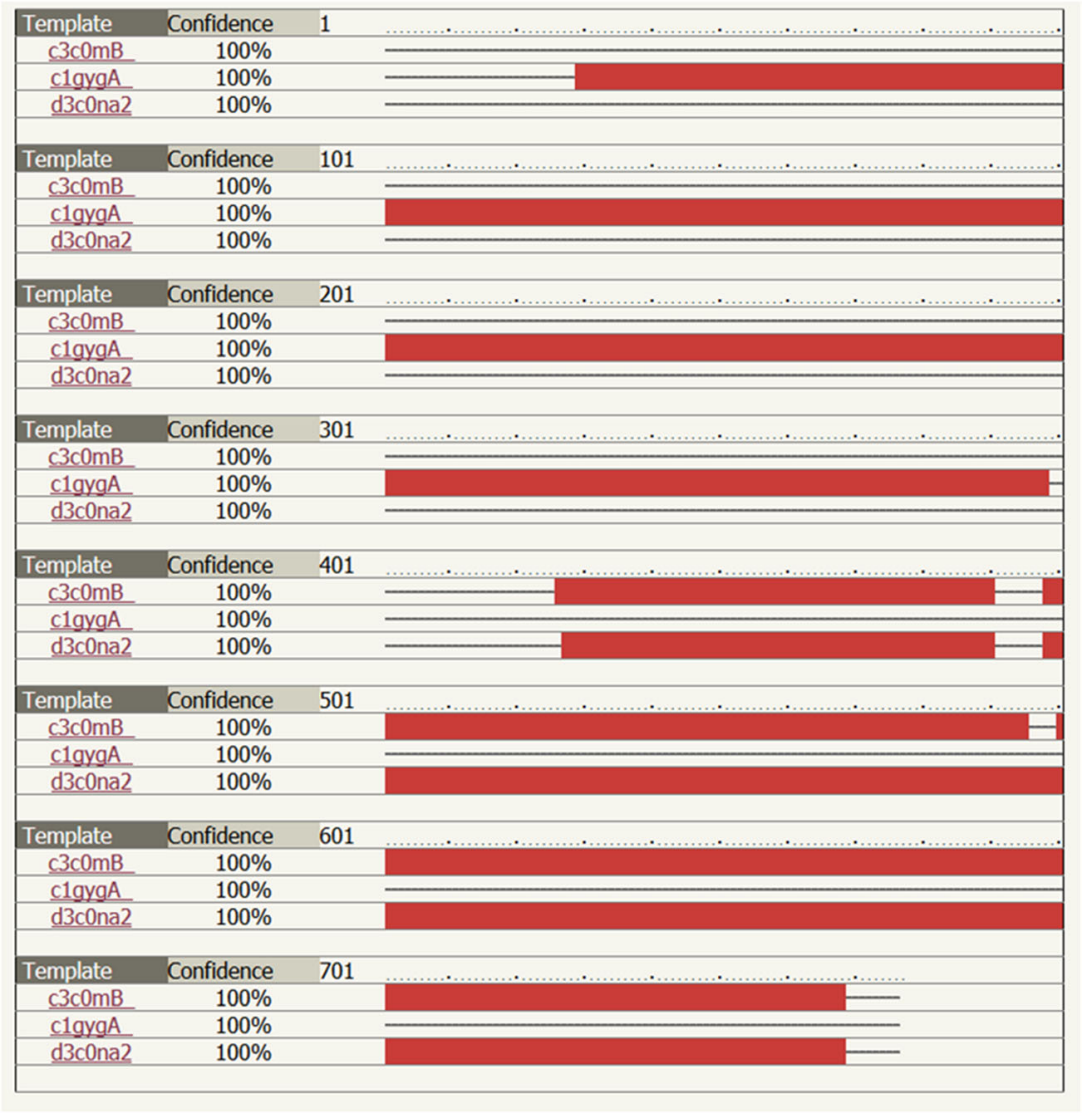
Predict potential binding sites of the fusion protein by 3DLigandSite server

Patterns and profiles of the construct fusion protein displayed that the α-α fusion protein are made up of *C. perfringens* alpha toxin/phospholipase C/P1 nuclease domain superfamily and PLAT/LH2 domain superfamily, the family member which is combined with *C. septicum* alpha toxin/Aerolisin/ETX pore-forming (Fig. 2). The secondary structure results revealed that total residues are 777 aa, which consists of 4 beta sheets, 3 beta hairpins, 2 beta bulges, 12 strands, 24 helices, 29 helix-helix interactions, 110 beta turns, and 39 gamma turns (Fig. 3). SignalP program showed that the signal peptide cleavage site is located at amino acids (aa) number 1 to 28 (Fig. 4). The linker fragment is located at amino acids number 399 to 410, because there is a helix pick in the position (Fig. 5). Amino acids 1 to 398 present the alpha toxin of *C. perfringens* type A, and amino acids 411 to 777 belong to the alpha toxin of *C. septicum*. HMMTOP program showed that the fusion protein N-terminus is IN, and transmembrane helices are located between amino acid number 8 and 27. Analysis of the α-α fusion protein, using the I-TASSER server, is revealed as C-score = − 2.68, TM-score = 0.41 ± 0.14, and estimated root mean square deviation (RMSD) = 15.1 ± 3.5, and the visualization tertiary structure of the fusion protein was used from UCSF chimera (Fig. 6). Physicochemical parameters result of the fusion protein sequence showed number of amino acids = 777 aa, molecular weight = 87,932.29 D, theoretical pI = 6.94, total number of negatively charged residues (ASP + Glu) = 109, total number of positively charged residues (Arg + Lys) = 107, formula = C_3932_H_6010_N_1048_O_1214_S_17_, and the estimated half-life is > 10 h (*E. coli*, in vivo). The instability index is calculated to be 21.87; this classifies the fusion protein as stable. Prediction of the fusion protein binding sites:

Analyzing the alpha-alpha fusion protein, using the 3 selected templates by phyre^2^ online software showed 91% of residues modeled at > 90% confidence. Figure 7 Confidence of alpha-alpha fusion protein tertiary structure, based on three templets.

### Energy minimize and validation of the fusion protein

**Fig. 8:**
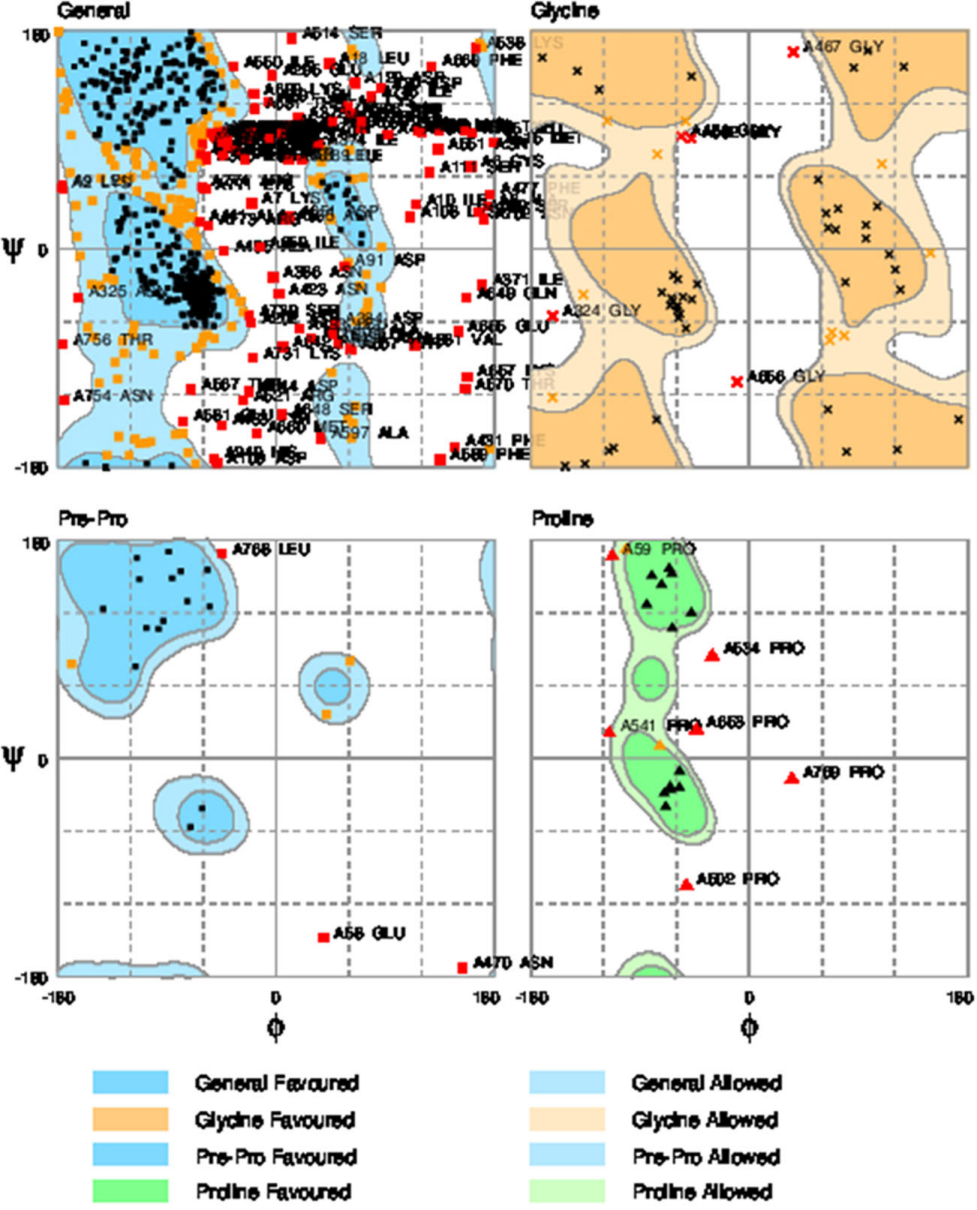
Ramachandran plot results of the α-α chimera fusion protein (777aa)

**Fig. 9:**
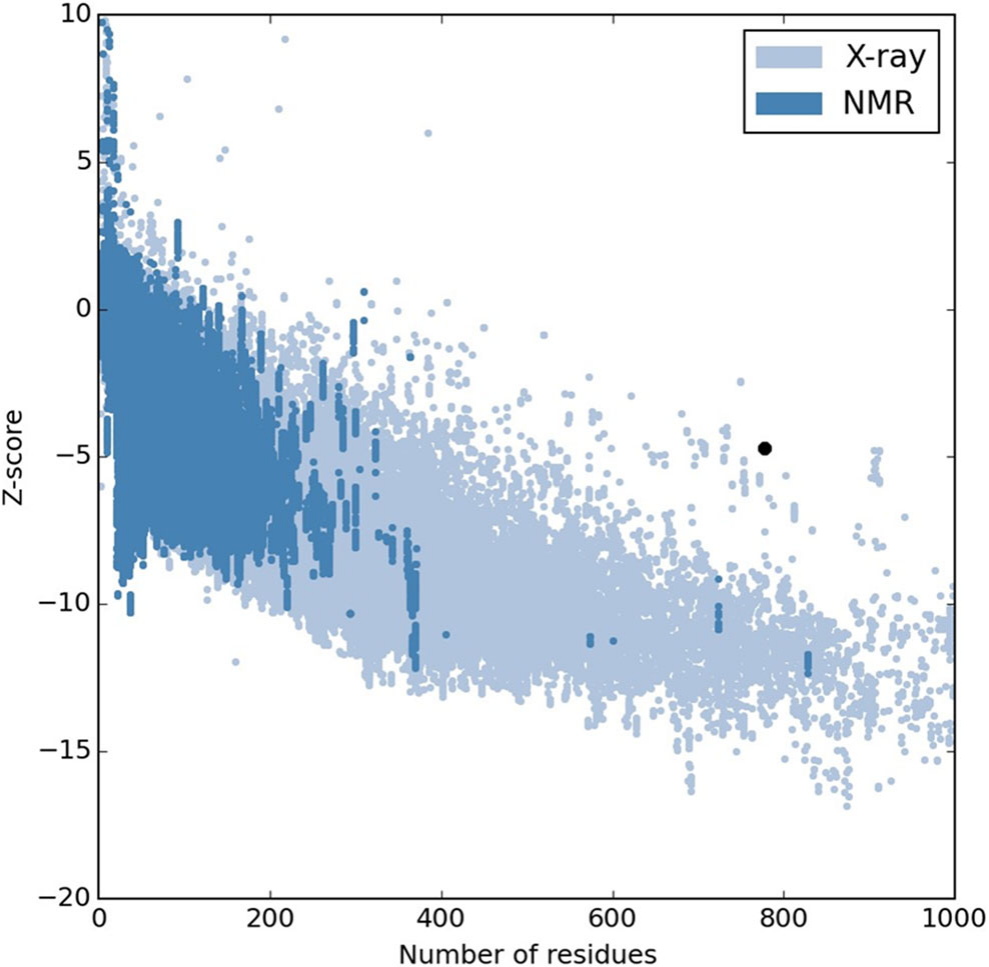
ProSA results of the fusion protein

Energy minimizing results of Chimera protein fusion showed E = − 26,991.951 kJ/mol. Ramachandran plot or Rampage software results showed that the synthetic alpha-alpha fusion protein model in the favored, allowed, and outlier regions have 70.5%, 17.9%, and 11.6% amino acid residues, respectively (Fig. 8). And also, ProSA software results showed: *Z*-Score = − 4.7 (Fig. 9). So, the results revealed that the structure of the fusion protein is valid. The deposited accession number for the sequence of the α-α fusion gene in the GenBank is MK908396.

## Discussion

Recombinant DNA technical methods have permitted fusions of genes in a simple way. The fusion of *cpa* and *csa* genes and its cloning in *E. coli* TOP10 has been reported (unpublished). In our work, in silico analysis of a chimeric fusion protein against *C. perfringens* type A and *C. septicum* alpha toxins is described. According to the newest findings, this is the 1st time that alpha-alpha fusion gene is designed for producing alpha-alpha fusion protein. It could be as a proper candidate for recombinant vaccine development. In our search, the complete *cpa* sequence containing its signal peptide for the proper secretion of the fusion protein and the *csa* sequence lacking signal peptide were used. A linker fragment “A(EAAAK)2A” was designed to link both genes (Langroudi et al. 2011). The InterPro program using several databases such as PRINTS, PROSITE, PFam-A, TIGRFAM, PROFILES, and PRODOM shows that the fusion protein consists of *C. perfringens* alpha toxin/phospholipase C/P1 nuclease domain superfamily and PLAT/LH2 domain superfamily, the family member which is combined with *C. septicum* alpha toxin/Aerolisin/ETX pore-forming (Jones et al. 2014). In 1989, Titball et al. showed that the nucleotide sequences of the *cpa* gene are 1197 bp in length (at base 1327–2523) and signal peptide sequences of the *cpa* gene are 84 bp in length (at base 1327–1410) and about 28 aa of that Trp may be the first amino acid of the mature activated toxin (Titball et al. 1989). In another search *csa* gene of *C. septicum*, BX96 was cloned and expressed in *E. coli*. The nucleotide sequences of the *csa* gene are 1332 bp in length (at base 561–1892) and signal peptide sequences of the *csa* gene are 93 bp in length (at base 561–653) about 31 aa (Ballard et al. 1995). The nucleotide sequences of the fusion protein displayed that the signal peptide site is located at amino acid number 1 to 28 (Fig. 4). For verification of this finding, the secondary structure and signal peptide of the fusion protein were predicted by proMotif of proFunc server (Laskowski et al. 2005a, b) and signalP (Petersen et al. 2011) online program, respectively. The data displayed similar features of each of the *C. perfringens* and *C. septicun* alpha toxin fragments comprising of alpha-alpha fusion protein constructions. The finding, which is shown in Fig. 6, verifies that the first residue of the fusion protein is amino acid Trp number 29. Based on the newest finding, for the 1st time, the designed fusion gene was constructed and cloned into pUC57 vector and then transformed into cloning host cell (*E. coli* TOP10) (unpublished). The deposited accession number for the sequence of the fusion gene in the GenBank is MK908396, and also, in GenBank protein ID QDK65251.1, there is translation of the fusion gene that would produce a 777 amino acid alpha-alpha fusion protein (Fig. 6). At the 5′ end of *cpa* and the 3′ end of *csa* were added *NdeI* and *XhoI* restriction sites and their flanking regions respectively, which are necessary for insertion of the fusion gene into pET22b (+) expression vector; they are in the synthetic construct alpha-alpha fusion gene. The InterPro results were verified by the findings of tertiary structure prediction of the fusion gene by I-TASSER server. Findings revealed that the fusion protein consists of two main domains linked together with a linker fragment. The secondary structure prediction results were verified by tertiary structure prediction findings of *cpa* complete alpha toxin, *csa*-activated alpha toxin, and five models of fusion proteins. The results displayed that the secondary structure characteristics of both toxins are present in model 1 of tertiary structure of fusion protein with the best C-score = − 2.68, TM-score = 0.41 ± 0.14 and RMSD = 15.1 ± 3.5 (Fig. 6). Different parts of the tertiary structure of the synthetic construct fusion protein were predicted by I-TASSER and are shown in (Fig. 6). The linker fragment between two domains of the fusion protein can provide proper flexibility and separation, and the fusion protein after expression can provide proper collection in the periplasmic space of suitable host cell, because a signal peptide is present at the N-terminal of fusion protein, which will permit it to cross the cytoplasmic membrane. Thus, the fusion protein will be secret to the culture media and inclusion body formation would not occur (Langroudi et al. 2012). I-TASSER server predicted models of protein by combining the methods of threading, structural refinement, and ab initio modeling (Roy et al. 2010; Zhang 2008; Zhang 2009). The final model in the procedure is created as PDB format. The model to the proFunc server upload and the server uses sequence and structure-based methods to determine the likely function of a protein from its 3D structure (Laskowski et al. 2005a, b).

ProtParam software was used to identify the physicochemical characteristics of the fusion protein sequence (Gasteiger et al. 2005). phyre^2^ software predicted the fusion protein binding sites (Kelley et al. 2015; Wass et al. 2010) and also Ramachandran plot and ProSA software results showed that the synthetic alpha-alpha fusion protein model is valid (Lovell et al. 2003; Wiederstein and Sippl 2007; Sippl 1993). In silico analysis is the most important approach to understand protein structure and functions. In conclusion, the designed fusion protein is valid and functional. Thus, the fusion gene could be used for clone and expression in a proper prokaryotic cell and also as a recombinant vaccine candidate.
